# Surgery for pilonidal sinus disease in Norway: training, attitudes and preferences—a survey among Norwegian surgeons

**DOI:** 10.1186/s12893-022-01889-1

**Published:** 2022-12-28

**Authors:** Mari Odlo, Julie Horn, Athanasios Xanthoulis

**Affiliations:** 1 Department of Clinical and Molecular Medicine, Norwegian University of Science and Technology, NTNU, Trondheim, Norway; 2 Department of Public Health and Nursing, Norwegian University of Science and Technology, NTNU, Trondheim, Norway; 3 Department of Obstetrics and Gynecology, Levanger Hospital, Nord-Trøndelag Hospital Trust, Levanger, Norway; 4 Department of Surgery, Levanger Hospital, Nord-Trøndelag Hospital Trust, Levanger, Norway

**Keywords:** Pilonidal sinus, Surgical procedures, Clinical competence, General surgery education, Survey, Norway

## Abstract

**Background:**

Pilonidal sinus disease (PSD) is frequently observed in young adults. There is no wide consensus on optimal treatment in the literature, and various procedures are used in clinical practice. The objective of this study was to assess current practice, experience, training, and attitudes towards PSD surgery among Norwegian surgeons.

**Methods:**

An online survey on PSD surgery was created and sent to all members of the Norwegian Surgical Association. Categorical data were reported as frequencies and percentages.

**Results:**

Most currently practicing Norwegian surgeons used the Bascom’s cleft lift (93.2%) or minimally invasive procedures (55.4%). Midline excisions with primary closure (19.7%) or secondary healing (22.4%) were still used by some surgeons, though. Most surgeons had received training in PSD surgery supervised by a specialist, but only about half of them felt sufficiently trained. The surgeons generally performed few PSD operations per year. Many considered PSD as a condition of low surgical status and this patient group as underprioritized.

**Conclusions:**

Our findings suggest that PSD surgery in Norway has been moving away from midline excisions and towards off-midline flap procedures and minimally invasive techniques. PSD and its treatment have a low status among many Norwegian surgeons. This study calls for attention to this underprioritized group of patients and shows the need for consensus in PSD treatment such as development of national guidelines in Norway. Further investigation on training in PSD and the role of supervision is needed.

## Background

Pilonidal sinus disease (PSD) is an inflammatory condition mainly affecting the sacrococcygeal area. The disease is frequently observed in young adults, predominantly males [1, 2]. The incidence ranges between 26 and 94 per 100,000, with an increase over the last decades [3, 4]. Besides male gender, predisposing factors include obesity, family history, hirsutism, a deep natal cleft, sedentary lifestyle, and local trauma [2, 5]. PSD can be asymptomatic, or present as an acute abscess in the gluteal cleft, or as chronic disease with intermittent discharging sinus(es) [5, 6]. Treatment options for chronic PSD include limited or wide excisions with secondary wound healing or primary closure in the midline or off-midline, possibly using tissue flaps [5]. Alternative treatment options are endoscopic procedures and other minimally invasive approaches [7, 8]. Prior studies have shown various long-term results depending on the choice of procedure, with higher recurrence rates for open healing and midline primary closure compared to off-midline procedures [9]. Nevertheless, there is no consensus on optimal treatment in literature. No surveys of current practice in Norway have been published in peer-reviewed literature. Furthermore, previous studies on PSD have mainly focused on outcomes of surgical techniques, but less is known on attitudes towards PSD and training in surgical treatment for PSD. Brown and Lund reported frequent use of simple excision techniques in the UK despite prolonged recovery, and suggested lack of interest in PSD surgery as a possible reason [10]. Similar findings from Denmark also indicated that surgeons may regard treatment of PSD as a low status activity [11]. Understanding uncertainties in current practice, experiences, and attitudes towards PSD surgery among Norwegian surgeons might impact training in PSD surgery as well as the development of national guidelines.

## Methods

A questionnaire was created for surgeons on the topic of PSD surgery by using nettskjema.no, a survey solution developed and hosted by the University of Oslo, Norway (nettskjema@usit.uio.no). It included questions about demographics, surgical training, and experience, preferred surgical procedures and attitudes towards PSD. The survey was conducted from June 1st to August 31st, 2021. The Norwegian Surgical Association (NKF) forwarded an e-mail containing a web-link to the questionnaire to all its members on behalf of the research team. A follow-up reminder was sent 2 weeks later. NKF is an umbrella association with members from all surgical specialties in Norway, both residents, specialists, and retirees. According to personal communication with NKF the proportion of gastrointestinal surgeons, general surgeons and other surgical subspecialities among NKF members is 19.1%, 40% and 40.9%, respectively. No information that could reveal personal details about the participants was collected. The Norwegian Centre for Research Data (NSD) assessed the study as not being subject to notification. This research project was designated as exempt from ethical review from the Regional Committee for Medical and Health Research Ethics (REC Central). Completion of the survey was considered to imply informed consent. Data analysis was performed using SPSS Statistics version 27 (IBM corp., Armonk, NY, USA). Categorical data were reported as frequencies and percentages.

## Results

**Fig. 1:**
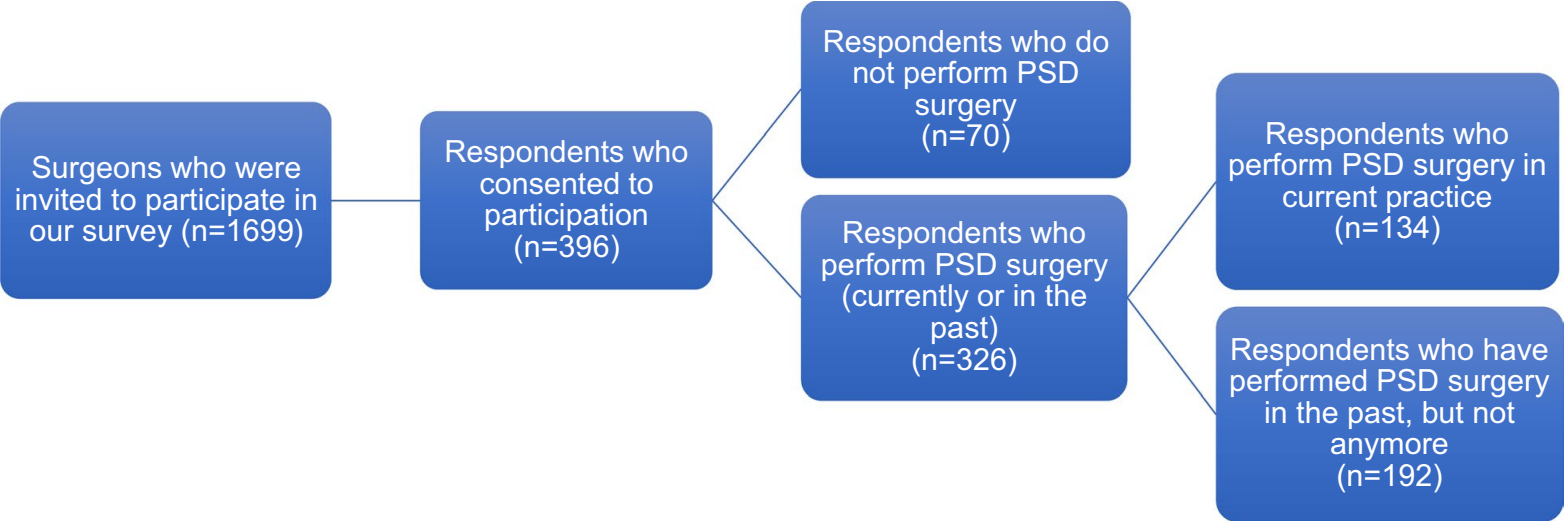
Survey recruitment process. *PSD* pilonidal sinus disease

Among 1699 invited surgeons, 396 consented to participation and completed at least parts of the survey (response rate: 23.3%). Of those, 70 participants did not perform PSD surgery, mostly because their respective field of surgery did not include PSD surgery (81.4%), but also due to lack of knowledge (5.7%) and/or experience (2.9%) (not shown). The remaining 326 participants who reported performing PSD surgery (currently or previously) proceeded with the rest of the survey and comprised our study population (Fig. 1).

Table 1 summarizes the respondents’ characteristics. Most respondents were specialists (48.6%), had more than 10 years of experience (58.5%), and worked at a public hospital (86%). All regions of Norway were represented with a predominance of South-Eastern Norway (49.8%), which reflects the population distribution in Norway. Among the 134 respondents who performed PSD surgery in current practice, 47% were residents, 57.2% had less than 10 years of experience, and 65.7% performed fewer than 10 PSD operations per year. However, 56.3% of the specialists and 70% of the surgeons with more than 10 years of experience no longer performed PSD surgery.

**Table 1:** Characteristics of survey respondents who perform surgery for pilonidal sinus disease

Respondent characteristics	All *n* = 326 (%)	Current practice^a^ *n* = 134 (%)	Previous practice^b^ *n* = 192 (%)
Position (*n* = 325)			
Residents	95 (29.2)	63 (47)	32 (16.8)
Specialists in general and/or gastrointestinal surgery	158 (48.6)	69 (51.5)	89 (46.6)
Other (e.g., retired)	72 (22.2)	2 (1.5)	70 (36.6)
Years of experience (*n* = 325)			
< 2	19 (5.8)	15 (11.3)	4 (2.1)
2–5	55 (16.9)	36 (27.1)	19 (9.9)
6–10	61 (18.8)	25 (18.8)	36 (18.8)
> 10	190 (58.5)	57 (42.9)	133 (69.3)
Number of PSD surgeries performed per year (*n* = 325)			
0–10	262 (80.6)	88 (65.7)	174 (91.1)
10–20	45 (13.8)	35 (26.1)	10 (5.2)
> 20	18 (5.5)	11 (8.2)	7 (3.7)
Practice type (*n* = 322)			
Public hospital	277 (86)	116 (86.6)	161 (85.6)
Private hospital	9 (2.8)	2 (1.5)	7 (3.7)
Both public and private hospital	36 (11.2)	16 (11.9)	20 (10.6)
Hospital trust/region^c^ (*n* = 323)			
South-Eastern Norway Regional Health Authority	161 (49.8)	68 (51.1)	93 (48.9)
Western Norway Regional Health Authority	45 (13.9)	13 (9.8)	32 (16.8)
Central Norway Regional Health Authority	60 (18.6)	27 (20.3)	33 (17.4)
Northern Norway Regional Health Authority	45 (13.9)	25 (18.8)	20 (10.5)
Other	22 (6.8)	2 (1.5)	20 (10.5)

*PSD* pilonidal sinus disease

^a^Respondents who currently perform surgery for pilonidal sinus disease

^b^Respondents who previously performed surgery for pilonidal sinus disease

^c^Respondents were able to choose multiple answers

### Surgical techniques

**Fig. 2:**
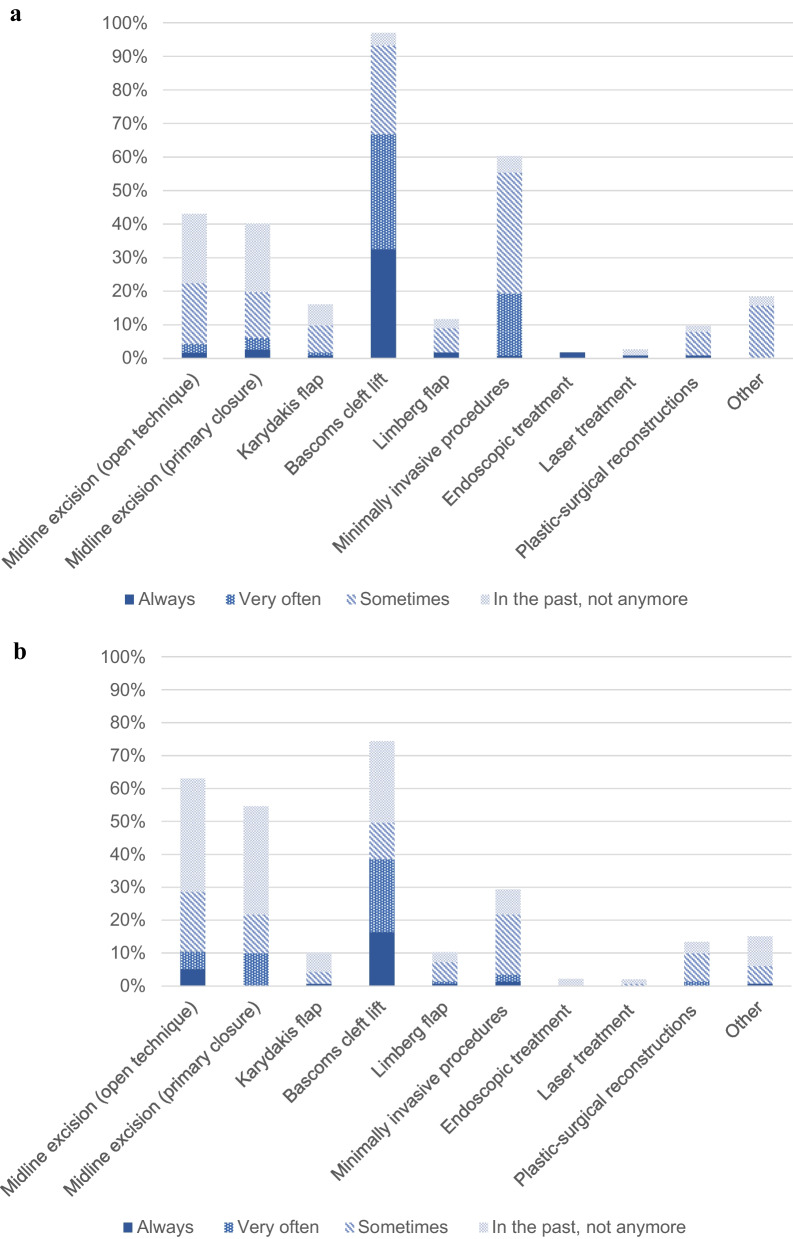
Surgical techniques for pilonidal sinus disease **a** among respondents who currently perform surgery for pilonidal sinus disease; **b** among respondents who previously performed surgery for pilonidal sinus disease

Figure 2 compares the surgical techniques used by surgeons performing PSD surgery. Bascom’s cleft lift was the predominant surgical technique among all respondents. Nearly all surgeons performing PSD surgery reported using Bascom’s cleft lift at least sometimes in current practice (93.2%). In contrast, 74.4% of the surgeons who no longer performed PSD surgery reported to have experience with this technique. Other off-midline flap procedures including Limberg flap and Karydakis flap were seldom practiced. Minimally invasive procedures (55.4%), such as modified Lord-Millar's procedure, Gips’ procedure, and Bascom’s pit picking represented the second most used procedure category among currently practicing surgeons. However, they were not as popular among the previously practicing surgeons. Endoscopic procedures were almost never practiced by either currently or previously practicing surgeons. Midline excisions with open technique or primary closure were used by respectively 63% and 54.6% of the respondents who no longer practiced PSD surgery. These techniques were less used by currently practicing respondents, with 22.4% performing midline excision with open technique and 19.7% midline excision with primary closure. Additionally, 20.7% (open technique) and 20.5% (primary closure) of the currently practicing surgeons reported to have used midline excisions before, but not anymore.

### Surgical training

Most respondents stated that they had received training in PSD surgery under the supervision of a specialist (83.3%) and/or a peer (28.1%). A total of 47.1% of the respondents considered themselves as sufficiently trained in PSD surgery. More than half of the respondents (61.3%), most of them specialists, instructed others in performing PSD surgery. Only 34.5% of the instructors reported following up their candidates consistently (Table 2).

**Table 2:** Training in surgery for pilonidal sinus disease

	*n* = 326 (%)
Which of the following training methods did you experience as part of your training in PSD surgery?^a^ (*n* = 324)	
Training supervised by a specialist	270 (83.3)
Training supervised by another resident	91 (28.1)
I am self-educated	58 (17.9)
I have been to a course	52 (16)
Other	37 (11.4)
I feel sufficiently trained in PSD surgery (*n* = 312)	
Agree/strongly agree	147 (47.1)
Neutral	62 (19.9)
Disagree/strongly disagree	103 (33)
I keep myself updated on the development of new treatments of PSD (*n* = 314)	
Agree/strongly agree	104 (33.1)
Neutral	53 (16.9)
Disagree/strongly disagree	157 (50)
Do you instruct others in performing PSD surgery? (*n* = 326)	
Yes, currently, or previously	200 (61.3)
Do you follow up your surgical candidates after instruction? (*n* = 197)	
Yes, always	68 (34.5)
Sometimes	94 (47.7)
No, never	35 (17.8)

*PSD* pilonidal sinus disease

^a^Respondents were able to choose multiple answers

Table 3 presents respondents’ attitudes and experiences according to their training in PSD surgery (supervision by a specialist vs. no specialist supervision). A greater proportion of the group without specialist supervision (43.1%) did not feel sufficiently trained compared to the group with supervised training by a specialist (30.8%). Compared to respondents supervised by specialists, those without specialist supervised training were less likely to state that PSD was discussed often at their department (10% vs. 22.7%) or that their department had a standard method for PSD surgery (37.7% vs. 56.2%) (not shown). In addition, respondents without specialist supervised training were more likely to experience recurrence and/or prolonged wound healing after PSD surgery (51% vs. 37.4%).

**Table 3:** Attitudes and experiences with surgery for pilonidal sinus disease according to respondents’ training

Survey statement (*n* = 316)	Agree/strongly agree (%)	Neutral (%)	Disagree/strongly disagree (%)
I feel sufficiently trained in PSD surgery (*n* = 311)			
Training supervised by specialist^a^ (*n* = 260)	47.3	21.9	30.8
No specialist supervision^b^ (*n* = 51)	47.1	9.8	43.1
I keep myself updated on the development of new treatments of PSD (*n* = 312)			
Training supervised by specialist (*n* = 261)	33.3	17.2	49.4
No specialist supervision (*n* = 51)	31.4	15.7	52.9
PSD surgery is simple and does not require specific training (*n* = 313)			
Training supervised by specialist (*n* = 262)	6.1	8	85.9
No specialist supervision (*n* = 51)	7.8	3.9	88.2
Patients with PSD are often underprioritized (*n* = 310)			
Training supervised by specialist (*n* = 260)	71.5	16.5	11.9
No specialist supervision (*n* = 50)	72	22	6
PSD and its treatment are considered to have low status compared to other surgical diseases among surgeons in general (*n* = 311)			
Training supervised by specialist (*n* = 261)	73.2	16.9	10
No specialist supervision (*n* = 50)	76	20	4
PSD and its treatment are considered to have low status compared to other surgical diseases at my department (*n* = 307)			
Training supervised by specialist (*n* = 256)	52.7	27.3	19.9
No specialist supervision (*n* = 51)	54.9	31.4	13.7
We often discuss PSD surgery at my department (*n* = 305)			
Training supervised by specialist (*n* = 255)	22.7	27.5	49.8
No specialist supervision (*n* = 50)	10	22	68
Performing PSD surgery feels meaningful to me (*n* = 307)			
Training supervised by specialist (*n* = 257)	82.5	13.6	3.9
No specialist supervision (*n* = 50)	84	16	0
Treating patients with PSD is often difficult (*n* = 307)			
Training supervised by specialist (*n* = 258)	62.4	20.2	17.4
No specialist supervision (*n* = 49)	67.3	22.4	10.2
I often experience recurrence/prolonged wound healing after PSD surgery (*n* = 303)			
Training supervised by specialist (*n* = 254)	37.4	29.1	33.5
No specialist supervision (*n* = 49)	51	32.7	16.3
I refer to another colleague/hospital if recurrence of PSD occurs (*n* = 302)			
Training supervised by specialist (*n* = 253)	29.6	33.2	37.2
No specialist supervision (*n* = 49)	38.8	18.4	42.9

*PSD* pilonidal sinus disease

^a^Respondents who have received training in pilonidal sinus disease surgery supervised by a specialist

^b^Respondents who have not received training in pilonidal sinus disease surgery supervised by a specialist, may have received training supervised by a peer and/or been to course(s) and/or been self-educated

### Attitudes

Most respondents agreed that patients with PSD are often underprioritized (71.4%), that PSD and its treatment most likely have low status compared to other surgical diseases among surgeons in general (73.5%), and at their department (52.9%). Still, respondents generally stated that performing PSD surgery felt meaningful (82.8%) and disagreed with the notion that PSD surgery is simple, not requiring special training (86.3%) (Table 4).

**Table 4:** The respondents’ attitudes and experiences with surgery for pilonidal sinus disease

Survey statement (*n* = 318)	Agree/strongly agree (%)	Neutral (%)	Disagree/strongly disagree (%)
PSD surgery is simple and does not require specific training (*n* = 315)	6.3	7.3	86.3
Patients with PSD are often underprioritized (*n* = 311)	71.4	17.7	10.9
PSD and its treatment are considered to have low status compared to other surgical diseases among surgeons in general (*n* = 313)	73.5	17.6	8.9
PSD and its treatment are considered to have low status compared to other surgical diseases at my department (*n* = 308)	52.9	28.2	18.8
We often discuss PSD surgery at my department (*n* = 306)	20.6	26.5	52.9
Performing PSD surgery feels meaningful to me (*n* = 309)	82.8	13.9	3.2
Treating patients with PSD is often difficult (*n* = 309)	62.8	20.7	16.5
I often experience recurrence/prolonged wound healing after PSD surgery (*n* = 305)	39.7	29.8	30.5
I refer to another colleague/hospital if recurrence of PSD occurs (*n* = 304)	30.9	30.9	38.2

*PSD* pilonidal sinus disease

## Discussion

In the present study, we investigated preferences, training, and attitudes towards PSD surgery among Norwegian surgeons. Most surgeons prefer Bascom’s cleft lift or minimally invasive procedures. Midline excisions are sometimes still performed, though. Only about half of the surgeons feel sufficiently trained in PSD surgery and many consider PSD as a condition of low surgical status and the patients as underprioritized.

Currently, only Italy, Germany, and the US have developed national guidelines for PSD treatment [12–14]. There is wide agreement that midline excisions with primary closure should be abandoned due to increased recurrence and wound dehiscence [12–16]. Yet, midline closure is still used at least sometimes by approximately 20% of the Norwegian surgeons in our study. Earlier studies from the UK and Ireland, Switzerland, Austria, and Denmark have reported even higher rates of midline closure, but this practice may have changed over the last years in favor of off-midline closure [11, 17, 18]. Our findings of lower rates of midline closure among currently practicing surgeons compared to surgeons no longer performing PSD surgery may indicate a decrease over the last years. Likewise, currently practicing surgeons appear to have changed their practice away from midline closure according to new recommendations.

A similar procedure is midline excision with secondary healing. A recent meta-analysis stated that this technique can be justified despite a 10-year recurrence rate of 20% [19]. However, despite its technical simplicity, open wound healing is associated with extended time off work and decreased postoperative quality of life [15, 20]. German and Italian guidelines are critical to open healing, while US guidelines present this as an option [12]. We observed that Norwegian surgeons tend to agree more with German and Italian guidelines, as use of midline excision with secondary healing seems to decline.

Off-midline procedures, such as Bascom’s cleft lift, Karydakis flap, and Limberg flap, are recommended because of better postoperative outcomes and lower recurrence rates [10, 12–14, 20, 21]. It has not been possible to identify the single best off-midline procedure [22–25]. While the Limberg flap and the Karydakis flap are preferred in many countries, we found that they are seldom used in Norway, where nine out of ten currently practicing surgeons reported using the Bascom’s cleft lift [16, 17, 26, 27]. A majority of the participating surgeons in our study reported to use flap procedures. This is in contrast to findings from a recent study that reported an infrequent use of flap techniques (22.6%) by Dutch surgeons [28]. It seems like most hospitals in Norway have followed the example of The University Hospital of North Norway, Tromsø, that introduced this technique as their standard procedure for all symptomatic, chronic PSD already in 2002 [29].

Minimally invasive procedures, such as modified Lord-Millar's procedure, Gips’ procedure, and Bascom’s pit picking, show various recurrence rates, but have the advantages of less pain, faster wound healing, and shorter time off work [12, 30, 31]. Minimally invasive techniques are recommended for limited disease without previous failed surgery [12, 13]. Similar to a study from Switzerland, we found that minimally invasive procedures were widely used in Norway [17]. The trend of fewer midline procedures in favor of more off-midline flap-surgery and minimally invasive procedures in Norway is welcome, and comparable to recent treatment strategies for PSD in Switzerland and Austria [17]. On the other hand, endoscopic procedures are seldom used, although studies have suggested that these are effective treatment modalities [8, 32].

One in six surgeons in our study has not been supervised by a specialist during his/her training. In addition, only one third of the instructors stated consistent follow up of their trainees. Accordingly, a survey in the UK and Ireland reported that 29% of the respondents had not received formal training in the surgical procedure of their choice in PSD management [18]. Similar to this study, we were not able to further assess the extent and quality of supervision. Only about half of the respondents considered themselves sufficiently trained in PSD surgery, especially those who had not been supervised by a specialist. This may suggest limited focus on PSD surgery and/or inadequate quality of supervision. Similarly, a recent survey among Danish surgical residents found that self-perceived readiness to perform surgery after completion of the surgical residency program was significantly associated with the level of supervision [33]. We found that surgeons supervised by a specialist during their training were less likely to experience recurrence and/or prolonged wound healing after PSD surgery. This may indicate that increased supervised training in PSD surgery leads to fewer recurrences. However, our findings do not reflect an objective difference in recurrence rate and/or prolonged wound healing, but rather respondents’ subjective estimation. These differences could have been influenced by other aspects, such as patient selection, experience, follow-up time, and choice of procedure.

In addition to adequate supervision, surgical outcome depends on surgeon’s volume [34]. Hopper et al. suggested that learning curves exist for all surgical procedures [35]. Wysocki has shown that competence using the modified Karydakis flap was achieved after case 10 to 21, and proficiency after case 30 to 51 [36]. Comparable learning curves may exist for the other treatment options for PSD. Currently, the specialization in general or gastrointestinal surgery in Norway does not require a specific number of PSD procedures performed. Like numbers from other countries, a large proportion of the surgeons performing PSD surgery in current practice performed fewer than ten PSD operations per year [11, 27, 28]. This indicates that it may take longer time to achieve competence in PSD surgery. PSD may be better addressed by a few interested surgeons with a larger surgical volume, as others have also suggested [37].

Album and Westin described a prestige rank order of diseases, where slowly developing diseases with long duration and diseases in the lower part of the body are given low prestige scores [38]. This agrees with our findings which showed that a high proportion of surgeons considered PSD as a low-status disease and the patient group as underprioritized. Similar to a study from Denmark, we found that PSD surgery was often performed by less experienced surgeons in an earlier phase of their career [11]. PSD was also seldom discussed among colleagues, especially in the group without specialist supervision in PSD surgery. Others have also suggested a lack of experience and/or interest in PSD among surgeons, further underlining the low prestige of PSD [10].

Few studies have investigated surgeons’ preferences in PSD surgery, and even fewer have examined surgical training and the status of the disease. In Norway, no similar study has been published. The present survey was answered by Norwegian surgeons treating PSD either previously or in current practice, giving the opportunity to compare procedures and attitudes among surgeons in the past and now. One drawback of the survey is the lack of distinction among respondents who no longer perform PSD surgery. This group includes both working surgeons and retirees, and we do not know when these surgeons stopped performing PSD surgery. When analyzing the results, it was clear that some of the questions were open to interpretation by the previously practicing group.

The overall response rate of the survey was 23.3%, which is slightly lower than typical response rates around 30–35% in previous web-based surveys answered by physicians [39, 40]. In our study, all members of the Norwegian Surgical Association were invited to participate. The association has members from all surgical specialties in Norway. Approximately 40% of the invited members do not perform PSD surgery as part of their specialty, which can explain the low response rate. However, our study population was representative with respect to geographical distribution, work position, and level of experience. Response rates may have been higher among surgeons with a greater interest in PSD surgery, possibly leading to selection bias. Interested surgeons may be more likely to follow evidence-based treatment strategies, and this can potentially contribute to an overestimation of the proportion of Bascom’s cleft lift and minimally invasive procedures in our study.

## Conclusions

Our findings suggest that PSD surgery in Norway has been moving away from midline excisions and towards off-midline flap procedures and minimally invasive techniques, with the Bascom’s cleft lift being the most commonly performed procedure. Nevertheless, the midline closure is still used too often considering the evidence supporting better treatment options. Surgeons generally perform few PSD operations per year, and only about half of the surgeons feel sufficiently trained in PSD surgery. Further investigation on training in PSD and the role of supervision is needed*.* PSD and its treatment have a low status among many Norwegian surgeons. This study calls for more attention to this less prioritized group of patients and shows the need for consensus in PSD treatment such as development of national guidelines in Norway.

## Data Availability

The dataset used and analyzed during the current study are available from the corresponding author on reasonable request.

## Abbreviation

PSD: Pilonidal sinus disease
